# Morphological and Spectral Features of Ionospheric Structures at E- and F-Region Altitudes over Poker Flat Analyzed Using Modeling and Observations

**DOI:** 10.3390/s23052477

**Published:** 2023-02-23

**Authors:** Pralay Raj Vaggu, Kshitija B. Deshpande, Seebany Datta-Barua, Gary S. Bust, Donald L. Hampton, Aurora López Rubio, James P. Conroy

**Affiliations:** 1 Department of Physical Sciences, Embry-Riddle Aeronautical University, Daytona Beach, FL 32114, USA; 2 Illinois Institute of Technology, Chicago, IL 60616, USA; 3 Johns Hopkins University Applied Physics Laboratory, Laurel, MD 21287, USA; 4 Geophysical Institute, University of Alaska, Fairbanks, AK 99775, USA; 5 Bradley Department of Electrical and Computer Engineering, Virginia Tech University, Blacksburg, VA 22043, USA

**Keywords:** ionospheric irregularities, radio wave propagation, irregularity spectra, auroral E- and F-regions

## Abstract

Electron density irregularities in the ionosphere modify the phase and amplitude of trans-ionospheric radio signals. We aim to characterize the spectral and morphological features of E- and F-region ionospheric irregularities likely to produce these fluctuations or “scintillations”. To characterize them, we use a three-dimensional radio wave propagation model—“Satellite-beacon Ionospheric scintillation Global Model of upper Atmosphere” (SIGMA), along with the scintillation measurements observed by a cluster of six Global Positioning System (GPS) receivers called Scintillation Auroral GPS Array (SAGA) at Poker Flat, AK. An inverse method is used to derive the parameters that describe the irregularities by estimating the best fit of model outputs to GPS observations. We analyze in detail one E-region and two F-region events during geomagnetically active times and determine the E- and F-region irregularity characteristics using two different spectral models as input to SIGMA. Our results from the spectral analysis show that the E-region irregularities are more elongated along the magnetic field lines with rod-shaped structures, while the F-region irregularities have wing-like structures with irregularities extending both along and across the magnetic field lines. We also found that the spectral index of the E-region event is less than the spectral index of the F-region events. Additionally, the spectral slope on the ground at higher frequencies is less than the spectral slope at irregularity height. This study describes distinctive morphological and spectral features of irregularities at E- and F-regions for a handful of cases performed using a full 3D propagation model coupled with GPS observations and inversion.

## 1. Introduction

The range and spread of satellite-based applications are growing fast due to the high efficiency, accuracy, and precision of Global Navigation Satellite System (GNSS) measurements. However, the fidelity of these measurements is limited by various factors, including the ionosphere [1,2,3,4]. There are three major regions of the ionosphere called the D-region (50–90 km), the E-region (90–150 km), and the F-region (approximately 150–500 km). These regions do not have a sharp boundary, and the altitude at which they occur varies day and night and from season to season [5,6]. Precipitation of energetic particles from the Sun and the Earth’s magnetosphere into the ionosphere modifies the ionospheric background electron density, thereby creating irregularities. The satellite signal propagating through the density irregularities of intermediate scale sizes (a few hundred meters–a few tens of kilometers) can cause radio signal fluctuations, henceforth referred to as ionospheric scintillations. The scintillation effects can be detrimental to GNSS signals, but they can be used as a diagnostic tool in studying ionospheric irregularities [7,8]. In this study, we utilize scintillation observations to examine the characteristic features of density irregularities over the auroral region using a forward electromagnetic (EM) wave propagation model and an inverse method.

In the auroral region, the density irregularities are subjected to auroral dynamics, particle precipitation, and plasma instabilities such as gradient drift instability (GDI), Kelvin Helmholtz instability (KHI), etc., generating density structures causing amplitude and phase fluctuations [9,10,11]. These disturbances in the auroral region showed a correlation with Global Positioning System (GPS) scintillation activity [12,13,14]. The enhancement of ionospheric scintillations is found at the boundaries of the auroral oval, and the auroral region shows more dominance of phase fluctuations than amplitude scintillations [8]. It is also found that the auroral scintillations frequently occur near midnight magnetic local time (MLT) and are strongly correlated with the disturbances in the local magnetic field [15], and possibly associated with precipitation during a substorm event [14]. We will look in detail at a few hand-picked phase scintillation events recorded at the auroral station that occurred during active geomagnetic times at E- and F-region altitudes, thereby analyzing the differences/similarities of irregularity morphology at E- and F-region ionosphere. The choice of the limited number of events is based on the availability of measurements from multiple SAGA receivers with at least 30 s long continuous scintillation and simultaneous measurements from other auxiliary data sets.

### 1.1. Background

The study of ionospheric scintillation and scintillation models is based on radio wave propagation theory in random media. Ionospheric density irregularities are usually described as a random medium, allowing to physically interpret the scintillation data in terms of the properties of the irregular ionosphere and the structures within [1,2]. These density irregularities distort the wavefront coming from the satellite, giving rise to a randomly phase-modulated wave. Further propagation towards the receiver causes further wave modulation, producing a complicated interference pattern on the ground. It is known that the fluctuations of a satellite radio signal are a consequence of the existence of random density irregularities within the ionosphere [1,2]. The spectrum of these irregularities can be measured in situ or estimated using phase and amplitude scintillations observed on the ground. The one-dimensional spectrum obtained from in situ probes can be effectively used to extract a three-dimensional spectrum by integrating over two dimensions. Such reconstruction is possible only for isotropic irregularities [16]. Since ionospheric irregularities are anisotropic, interpretation of spectra necessarily involves the information about the shape of the irregularities [17]. Previous studies on the anisotropy measurements of ionospheric irregularities indicate three general shapes of high latitude irregularities classified as rods, sheets, and wings [18,19,20]. The irregularities with sheet-like structures were observed during auroral precipitation in the F-region of auroral oval [19,21]. The nonlinear evolution of plasma instabilities explains the anisotropy of sheet-like irregularity structures in the auroral F-region [22,23,24]. The irregularity morphology in the auroral E-region is related to the current flows in the electrojet that causes rod-like field-aligned structures [25,26]. The irregularity spectrum of these density structures can be characterized by spectral models such as Hybrid [27] and Shkarofsky [1]. The spectral index is the power-law index of the irregularity spectra that can be dependent on the scale sizes of density irregularities. The spectral index computed for high-latitude F-region irregularities with scale sizes of 300 m to 1 km is 4 ± 1 [17]. Spectral representation of ionospheric density irregularities includes representation in terms of wave number or outer scale [28]. The outer scale ($l_{0}$) is defined as the largest spatial dimension of the irregularities, given by $l_{0}=2\pi /k_{0}$, where $k_{0}$ is the outer scale wave number. The spectral index of 4 is consistent for F-region measurements with outer scale wavenumber ($k_{0}$ = 2 km^−1^) [28]. The spectral indices can be different for irregularities at different altitudes. For example, the spectral index values for high latitude F-region irregularities vary from 2.5 to 4.5, whereas the E-region spectral index values range from 1.5 to 3.2 [29]. Despite the studies mentioned here, a comparison between auroral E- and F-region irregularity spectra and the irregularity shapes had been missing, which motivates this study.

### 1.2. Motivation

The spectral and morphological studies of high-latitude ionospheric irregularities have often been performed using satellite observations. A two-dimensional (2-D) analytic phase screen model is used to characterize the structures associated with a series of polar cap patches [30]. However, detailed observation and modeling-based study to understand auroral E- and F-region irregularity spectra and irregularity shapes using a phase screen three-dimensional (3-D) propagation model coupled with GNSS scintillation observations has not been explored. The motivation of this study is to analyze the similarities and/or differences between the E- and F-region irregularity morphological and spectral features in the ionosphere. We address this using a full 3-D global EM wave propagation model called “Satellite-beacon Ionospheric scintillation Global Model of the upper Atmosphere” (SIGMA) [31]. We investigate the spectral characteristics and the anisotropy of the ionospheric irregularities for three events that occurred at auroral E- and F-region altitudes during geomagnetically active times. The SIGMA model propagates the radio signal through the phase screens. A phase screen is assumed to introduce random signal phase fluctuations proportional to the electron density in that layer [1]. The spectral models Hybrid and Shkarofsky are used to characterize the distribution of ionospheric density irregularities in SIGMA and further study their morphology. An inverse method, as described by [32], is used to derive irregularity parameters such as root-mean-square (RMS) of the electron density fluctuations, spectral index, irregularity axial ratio, and drift velocity by comparing GNSS observations to the SIGMA outputs, which gives an insight into the physics of the irregularity structures involved in the generation of these GNSS scintillations.

The structure of this paper is as follows: In Section 2, we describe the observational data taken from the array of GPS receivers, auxiliary data, and the methodology of our analysis. We present the results obtained from our inverse analysis and irregularity shape sensitivity study in Section 3. We discuss the results in Section 4 and conclude the work in Section 5.

## 2. Data and Methodology

In this section, we describe the GPS measurements and auxiliary observations used in this study. We also discuss the model configuration and approach to incorporate the observational data into the model for further analysis and inversion.

### 2.1. Observational Data

Scintillation Auroral GPS Array (SAGA) has six Connected Autonomous Space Environment Sensor (CASES) GPS receivers established at Poker Flat Research Range (PFRR), Alaska [33]. Each SAGA receiver provides 50 Hz power and phase measurements for each satellite being tracked at L1 C/A (1575.42 MHz) and L2C (1227.60 MHz) frequencies (in this work, only L1 is used). We use the high rate time series data from CASES receivers, detrended and filtered as recommended by [34]. Here, the authors would like to acknowledge that there are different filtering techniques to determine the cut-off frequency for the filter in order to separate refractive vs. diffractive components of scintillation [35]. However, we are interested in phase fluctuations caused by ionospheric structures responsible for refractive and/or diffractive effects. Hence, 0.1 Hz high-pass filter cut-off suffices and is a convenient pick for an inverse method with thousands of filtered time series.

We examine the phase fluctuation events detected by SAGA, identified as E- or F-region events by the simultaneous Poker Flat Incoherent Scatter Radar (PFISR) measurements of electron density [36]. In their work, the height of the irregularity layer associated with each scintillation event is hypothesized to be due to the activity in the E-layer (below 150 km) or the F-layer (above 195 km) using PFISR density measurements. An event is an E-region event when its peak density is at E-layer altitudes and an F-region when the peak density is at F-layer altitudes. Though we estimate the irregularity height from the PFISR electron density measurements, we would like to emphasize that we also perform an inverse analysis at both (E- and F-region) heights for each event within SIGMA. By doing so, the event is marked as E-/F-region not only by looking at the peak electron density but also from the SIGMA inverse analysis. In this paper, the optimal irregularity heights obtained from inverse analysis for all events match the irregularity heights detected by the PFISR peak electron density (plots added as supplementary material). It is also reported that most events analyzed during 2014 and 2015 by [36] at PFRR are phase-only scintillation events.

**Figure 1:**
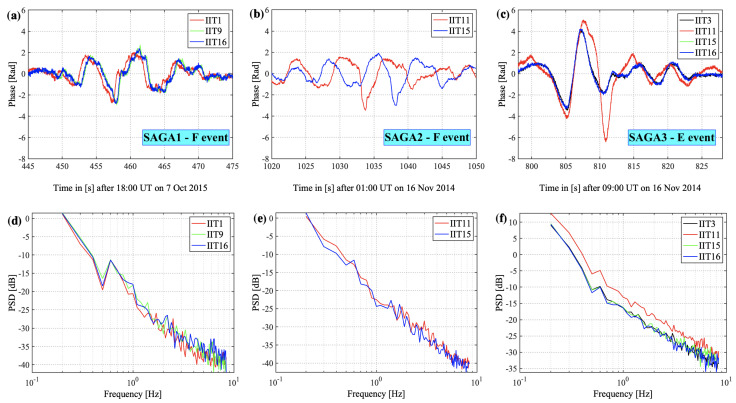
Time series of detrended and filtered GPS signal phase ($\Phi {}_{f}$) for L1 C/A, (**a**) PRN 25, an F-region event on 7 October 2015, (**b**) PRN 1, an F-region event, and (**c**) PRN 30, an E-region event, both on 16 November 2014. Data from all available SAGA receivers (IIT1, IIT3, etc.) during the scintillation interval are over-plotted for each event. (**d**–**f**) are the corresponding power spectral densities (PSD) for each event.

**Figure 2:**
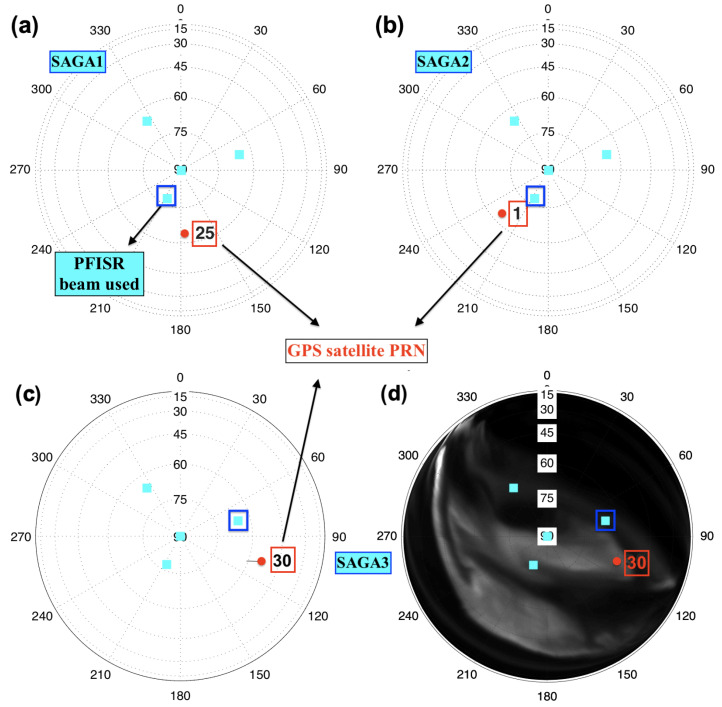
The sky plots of PFISR beam configuration (cyan squares) and the GPS satellites (solid red dots) (**a**) PRN 25 for SAGA1 event, (**b**) PRN 1 for SAGA2 event, and (**c**) PRN 30 for SAGA3 (satellite path with a solid red dot marked as endpoint) events. (**d**) Skyplot of PFISR beams and PRN 30 location for the SAGA 3 event is plotted over an all-sky image of 557.7 nm green line auroral emission during the scintillation interval.

The selected events for this study are—(1) an F-region event occurred on 7 October 2015 at 18:07:25 UTC (07:19 MLT) with geomagnetic activity index Kp 7 [37] (referred to as “SAGA1”), shown in Figure 1a; (2) another F-region event on 16 November 2014 at 01:17:00 UTC (14:20 MLT) with Kp index 4 (“SAGA2”), shown in Figure 1b; and (3) an E-region event on 16 November 2014 at 09:13:18 UTC (22:35 MLT) with Kp index 4 (“SAGA3”), shown in Figure 1c. For modeling studies, the SAGA receiver IIT9 (65.12${}^{\circ }$ N, −147.47${}^{\circ }$ E) is used for the SAGA1 event, and IIT11 (65.11${}^{\circ }$ N, −147.46${}^{\circ }$ E) is used for SAGA2 and SAGA3 events. The choice of the receiver is made based on the intensity of the observed phase fluctuations; strong phase fluctuations are selected for further modeling analysis. The phase fluctuations shown in Figure 1a–c are plotted for the SAGA receivers with continuous 50 Hz data during the scintillation interval. Data from all available receivers are used to obtain drift velocity estimates using the spaced-receiver method, which uses high-rate detrended, filtered phase measurements observed by multiple SAGA receivers [38]. We want to emphasize that the neutral wind dynamics at E-region heights would affect the SAGA drift estimates and are not considered in the current multi-receiver drift estimation technique. Most of the SAGA estimates encompass the F-region drifts, and for E-region drift estimates, we rely on the observations from PFISR. PFISR has an electronically steerable phased array capable of forming beams for different experiment modes [39]. The PFISR data we use have an experimental configuration of four beams, as shown in Figure 2. The configuration includes a zenith beam, a southward beam that points antiparallel to the local magnetic field, and two other beams spread out at similar elevation angles.

We look at the closest PFISR beam to the selected GPS satellite over Poker Flat. For F-region events, we consider the anti-parallel beam (beam at magnetic zenith) with 205.7${}^{\circ }$ azimuth and 77.5${}^{\circ }$ elevation (see Figure 2), the southernmost beam to PFISR. The beam with 76.1${}^{\circ }$ azimuth and 66.2${}^{\circ }$ elevation is used for E-region electron number density. The sky plot in Figure 2a–c shows the proximity of the GPS satellites and the PFISR beams used in this study. It shows that the signal fluctuations for SAGA1, SAGA2, and SAGA3 cases observed for the GPS satellites PRN 25, PRN 1, and PRN 30, respectively, can be seen close to the selected beams of PFISR. Figure 2d shows the all-sky image of the auroral arc plotted over the sky plot with the position of the PFISR beam and GPS satellite during the SAGA3 scintillation event. This indicates that the scintillation activity is more likely to correlate with the auroral activity evident from green line auroral emissions. SAGA1 and SAGA2 events occurred during local daylight, so no camera data are available.

### 2.2. Modeling

We use SIGMA in this study, a 3D forward propagation model that simulates the propagation of a radio wave through the ionospheric irregularities where the density distribution is represented by one or multiple phase screens or layers. We use spatial electron density distribution from the spectral models, Hybrid [27] and Shkarofsky [1] to generate the phase screens in SIGMA. The Hybrid spectrum in the irregularity coordinate system (in primed coordinates) is defined as

(1) $${P^{\prime }}_{NH}\left(\vec{k^{\prime }}\right)=\frac{a(\gamma_{H}-2)}{2\pi^{3/2}{k_{0}}^{2}}\Delta N^{2}{\left(1+\frac{{k_{x}{\;}^{\prime }}^{2}+{k_{y}{\;}^{\prime }}^{2}}{{k_{0}}^{2}}\right)}^{-\gamma_{H}/2}\exp\left(-{\left(ak_{0}\right)}^{2}\frac{{k_{z}{\;}^{\prime }}^{2}}{{k_{0}}^{2}}\right)$$

where $\gamma_{H}$ is the spectral index, ak_0_ depicts the axial ratio (AXR). ${k_{x}}^{\prime }$, ${k_{y}}^{\prime }$, and ${k_{z}}^{\prime }$ are the wave numbers in $x^{\prime }$, $y^{\prime }$, and $z^{\prime }$, respectively. $z^{\prime }$ and ${k_{z}}^{\prime }$ are along the magnetic field $B_{0}$, while the other two coordinates are in a plane perpendicular to $B_{0}$. *k*${}_{0}$ is the wave number associated with the outer scale *l*${}_{0}$. ΔN is the root-mean-square (RMS) of the electron density fluctuations, which are assumed to be generated by a zero-mean stationary random process. In the Hybrid spectrum, the irregularity distribution follows a power law in a plane perpendicular to the magnetic field and a Gaussian law along the magnetic field direction. The field lines are filled with plasma due to ionization in the auroral oval allowing the plasma distribution to be more like a Chapman distribution which could explain the Gaussian distribution along the field lines [27]. The symmetric nature of the Hybrid spectrum with respect to the magnetic field direction makes it suitable for modeling rod-like irregularities. Unlike the Hybrid, the Shkarofsky spectrum is a more generalized irregularity spectrum with a power-law distribution in all three directions that can be changed independently, allowing one to simulate rods, wings, and sheets for irregularity morphology. The Shkarofsky spectrum is given by

(2) $${\Phi^{\prime }}_{\xi }\left(\vec{k^{\prime }}\right)=\frac{{\sigma_{N}}^{2}{\left(k_{0}r_{0}\right)}^{(p-3)/2}{r_{0}}^{3}}{{\left(2\pi \right)}^{3/2}K_{(p-3)/2}\left(k_{0}r_{0}\right)}{\left(r_{0}\sqrt{{k^{\prime }}^{2}+{k_{0}}^{2}}\right)}^{-p/2}K_{\left(p/2\right)}\left(r_{0}\sqrt{{k^{\prime }}^{2}+{k_{0}}^{2}}\right)$$

where ${\sigma_{N}}^{2}$ is the variance of the electron density fluctuation, *p* is the spectral index, $r_{0}$ is the inner scale, $l_{0}=2\pi /k_{0}$ is the outer scale, $k_{0}$ is the wave number associated with the outer scale. $k^{\prime }$ is the spatial wave number defined as ${k^{\prime }}^{2}={a_{x}}^{2}{{k_{x}}^{\prime }}^{2}+{a_{y}}^{2}{{k_{y}}^{\prime }}^{2}+{a_{z}}^{2}{{k_{z}}^{\prime }}^{2}$ where $a_{x}$, $a_{y}$, and $a_{z}$ are the axial ratios in $x^{\prime }$, $y^{\prime }$, and $z^{\prime }$, directions, respectively. $K_{\mu }$ is the Bessel function of imaginary argument with order μ. The shape of irregularities is defined with the axial ratio elongation factors *a*${}_{x}$, *a*${}_{y}$, *a*${}_{z}$, which are the irregularity elongation factors in the geomagnetic east–west, in the geomagnetic north–south, and along the geomagnetic field line directions, respectively. Structures that extend in the direction of the magnetic field are referred to as rods, and their axial ratios are denoted as 1:1:*a*${}_{z}$ (*a*${}_{z}$> 1). Similarly, the structures extending both in magnetic east–west and along the field line direction are referred to as wings or sheets. The axial ratios for wings are *a*${}_{x}$:1:*a*${}_{z}$ (*a*${}_{x}$< *a*${}_{z}$), while the axial ratios *a*${}_{x}$:1:*a*${}_{z}$ (*a*${}_{x}$ = *a*${}_{z}$) denote the sheets. The Shkarofsky spectrum enables the irregularity distribution to be shaped independently in all three directions, whereas the Hybrid spectrum is interdependent in the x and y directions. Furthermore, note that Shkarofsky only has power-law distribution, while Hybrid can simulate Gaussian distribution for the spectral representation of irregularities. This study does not intend to compare these two spectral models as they have different density spectra; instead, we use both models to examine the irregularity characteristics to find the most suitable spectral representation of irregularities for each event under study.

**Figure 3:**
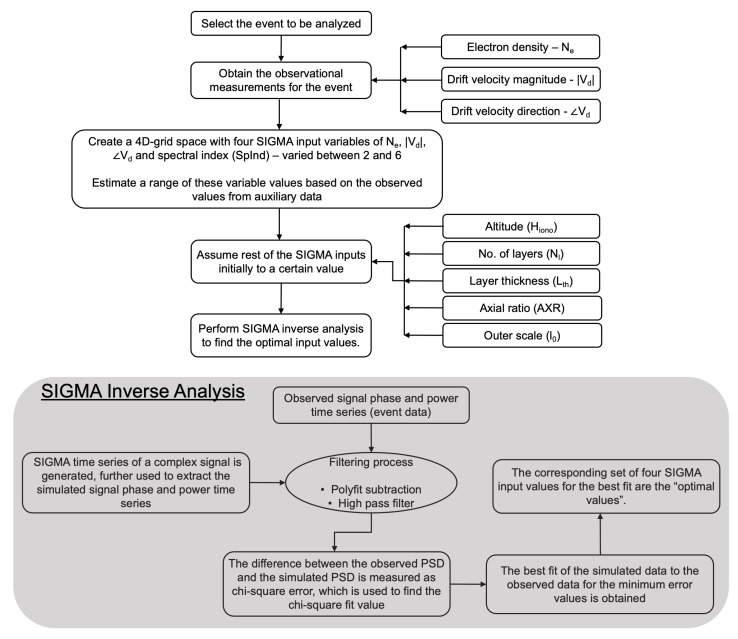
Flow chart illustrates the SIGMA process to find the optimal input values. The number of irregularity layers used in this study is one ($N_{l}$ = 1). We perform inverse runs with SIGMA at two different heights—one at 120km (E-region) and another at 350km height (F-region). We convert the outer scale ($l_{0}$) to outer scale wave number ($k_{0}$) within SIGMA. We fix the inner scales ($r_{0}$) to satisfy the Shkarofsky assumption that $k_{0}r_{0}<<1$ [1].

Figure 3 illustrates the process we adopt in analyzing an event with an inverse method and SIGMA to find the best-fit input values that best match the simulated power spectral density (PSD) to the observed PSD [32,40]. The process starts with selecting a scintillation event (phase and/or amplitude) detected by SAGA. The approximate observational values for SIGMA inputs such as electron number density (N_*e*_), drift speed $\left(|v_{d}|\right)$, and drift direction ($∠v_{d}$) for a selected event are obtained from PFISR and SAGA. We perform SIGMA simulation over a 4D-grid space with four design variables, namely, N_*e*_, $|v_{d}|$, $∠v_{d}$ and spectral index (SpInd), in addition to other five input parameters, namely, altitude ($H_{iono}$), no. of layers ($N_{l}$), layer thickness ($L_{th}$), axial ratio (AXR), and outer scale ($l_{0}$), which we do not vary during one inverse run. In this study, we only use one irregularity layer, and the $N_{l}$ parameter is not varied during the sensitivity analysis (discussed further). The initial range of values for the three design variables, namely, $N_{e}$, $|v_{d}|$, and $∠v_{d}$, are obtained from the auxiliary data. We consider a range of spectral index values from 2 to 6.

A high-rate (50 Hz) time series of a simulated complex signal is generated for each set of parameters, further used to extract the filtered signal phase and power time series. The observed power and phase time series data from SAGA and the simulated one for the selected event are detrended to remove the satellite geometry effects and receiver clock errors. We use the chi-square fitting test [41] to find the best PSD fit of the simulated data to the observed data. The maximum likelihood estimate of the model parameters is obtained by minimizing the chi-square quantity given by the equation below.

(3) $$\chi^{2}=\frac{1}{{\sigma_{y}}^{2}}\;\sum_{i=1}^{N}\;{\left(\log_{10}\;Y_{i}\;-\log_{10}\;X_{i}\right)}^{2}\;$$

where $\log_{10}Y_{i}s$ are N number of points on the PSD (dB) of the observed phase, $\log_{10}X_{i}s$ are the points on PSD (dB) of the SIGMA phase, and ${\sigma_{y}}^{2}$ is the variance on the observed PSD after removal of any trends. For a good fit [41], $\chi^{2}$≈ (N − M) or $\chi^{{}^{\prime }}$ = $\chi^{2}$/(N − M) ≈ 1, where the degrees of freedom are represented by (N − M), with M as the total number of design variables to be fitted. We vary the four design variables to evaluate the chi-square values at each grid point and find the minimal value. We also look at the confidence levels (later explained in Section 3) associated with the fit for the four design variables. The confidence intervals can be used to quantify ambiguities for the inverse runs we perform. The best fit obtained gives the optimal values for the four design variables, thereby providing a set that best describes the irregularity physics for the event under consideration. A sensitivity analysis (not mentioned in Figure 3) is further performed by keeping the optimal values of four design variables fixed and varying the remaining four SIGMA input parameters one at a time. This sensitivity analysis helps us gauge how sensitive the other four input parameters (which we did not iterate over during the inverse analysis) are to the model outputs. Estimating the sensitivities is a workaround to a higher (more than four) dimensional optimization problem with a large parametric space.

## 3. Results

**Figure 4:**
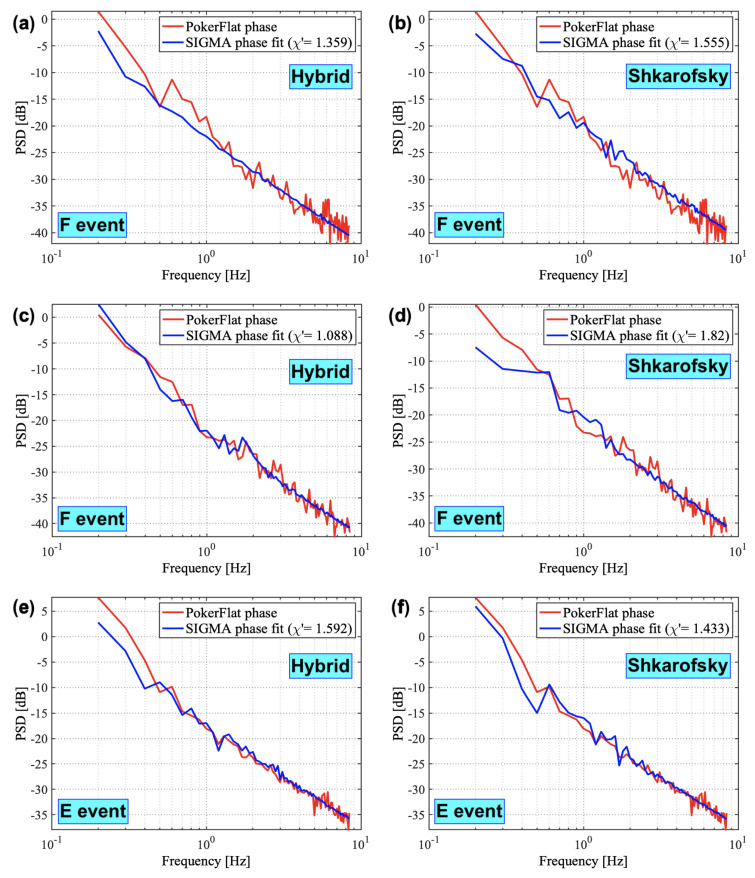
The observed and simulated PSD best fits for both Hybrid and Shkarofsky spectral models for the three events.

**Figure 5:**
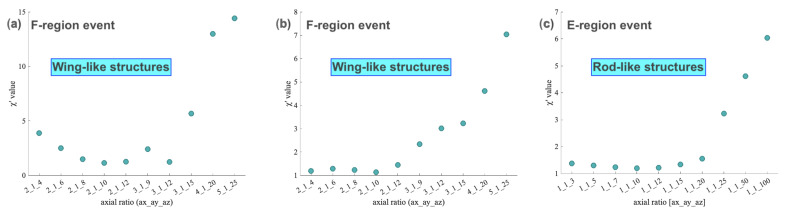
The SIGMA sensitivity analysis using the axial ratio parameter for all three events. The irregularity axial ratio representing wing-like structures fits well for F-region events and the axial ratio that represents rod-like structures best fits the E-region event.

This section presents results from our methodology described in Section 2 for all three events. Figure 4 represents results from the inverse analysis where we found the best fits for PSDs. Figure 4a,c,e show the best fit obtained for Hybrid spectrum. Figure 4b,d,f show the best fit obtained for Shkarofsky spectrum. The optimal values of the input parameters obtained for the best fit for all the events using both Hybrid and Shkarofsky spectra are mentioned in Table 1 and Table 2. $\chi {}^{\prime }$ values in PSD plots obtained by the method described in Section 2 shows how well the simulated PSDs fit the observed PSDs. $\chi {}^{\prime }$ values close to 1 are considered good fits. Figure 4a–d represent the F-region events at 350 km height, and Figure 4e–f is the E-region event at 120 km height identified by SAGA (as described in Section 2). The authors would like to mention that we performed the sensitivity study (discussed further) by varying the height of the irregularity layer to analyze the events at both the F-region and the E-region heights. Our analysis shows that E- and F-region event PSDs fit well when analyzed at SAGA-identified heights (Figure not shown). The best-fit input values for E- and F-region can be used for a comparative study of E- and F-region irregularities. The best-fit values obtained from the inverse analysis and the sensitivity study (discussed further) for SIGMA propagation parameters are shown in Table 1 and for SIGMA spectral parameters are shown in Table 2. The spectral parameters that include outer scale, axial ratio, spectral index, and RMS electron density fluctuation are used in spectral models. We call these spectral parameters for SIGMA. The propagation parameters, namely, the drift velocity, the height of the ionosphere, and the irregularity thickness are used in the forward propagation equation as explained by [31]. The propagation parameters shown in Table 1 include the values obtained from the PFISR and SAGA observations (shown under the observed column) along with the optimal values derived from SIGMA inverse analysis using Hybrid and Shkarofsky spectrum (shown under the simulated column). Table 2 values are obtained from the inverse analysis followed by a sensitivity analysis. We perform an inverse analysis with a range of values for four design variables and fix all other parameters, including the axial ratio parameter. We repeat this process for three different (fixed) axial ratios (one in the domain of the rods-like, one as wings-like, and one as sheets-like structure), for each of the three events, in order to see which is the best one, shown in Table 3. We then do a sensitivity analysis by varying the numerical values of axial ratios (elongation) for the same type of the given irregularity structure (rods/wings/sheets), shown in Figure 5. The best-fit values estimated are shown in Table 2. The simulated optimum values of electron density and drift velocity using both spectral models are within the range of PFISR observations for all cases. Similarly, the simulated optimum values of drift velocity agree well with the SAGA velocity estimates for F-region events.

**Figure 6:**
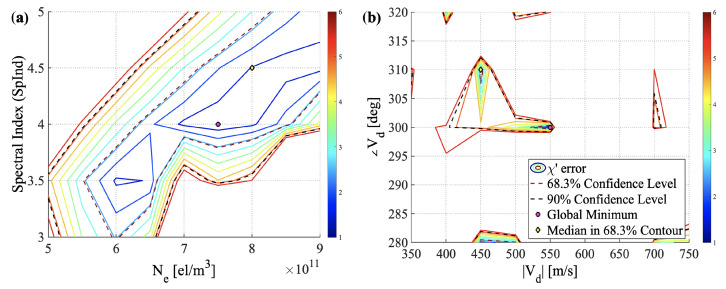
Contours showing the confidence levels of four SIGMA design variables for the SAGA2 event.

As stated earlier, no drift estimates from SAGA are available for E-region events because the frozen-in drift assumption does not hold at E-region heights. The best-fit selection is not just dependent on the $\chi {}^{\prime }$ fit but the confidence intervals as well. We identify confidence intervals at 68.3% and 90% (1 and 2 standard deviations) for different combinations of the four design variables. We discard cases when the solution appears on the edges of the parametric space to avoid ambiguity. Figure 6 shows an example of the confidence intervals for the SAGA2 event.

We present the contours of $\chi {}^{\prime }$ with respect to N_*e*_ and SpInd in Figure 6a and with respect to $|v_{d}|$ and $∠v_{d}$ in Figure 6b. In these plots, we show the position of the global minimum ($\chi {{}^{\prime }}_{min}$) (magenta circle) and the median (yellow diamond) in the units of the standard deviation spread. Dashed lines indicate the confidence level contours, and the solid lines indicate the contours of $\chi {}^{\prime }$ values. The contour level associated with $\chi {}^{\prime }$ values close to 1 (area covered under the solid dark blue contour) shows the parametric space where the model simulations (simulated PSD) have a close match with the observations (observed PSD). The 90% confidence level contour indicated by the black dotted line shows a 90% chance that the true values of the design variables will fall within that region. A multi-dimensional optimization problem may have ambiguities associated with the solutions. These confidence interval plots are used to quantify ambiguities for the inverse runs we have performed.

Following the inverse analysis, we perform a sensitivity analysis by keeping the optimal values of the four design variables fixed and varying the SIGMA input parameters (axial ratio, outer scale, and layer thickness) one at a time. We include the sensitivity analysis results by varying the irregularity axial ratio parameter. Here the author would like to note that we initially perform the inverse analysis for three different (fixed) axial ratios (one in the domain of the rods-like, one as the wings-like, and one as the sheets-like structure), for each of the three events to see which one best fits, shown in Table 3. We then perform a sensitivity analysis by varying the units of elongation of axial ratios (numerical values of the axial ratios) inside the domain of the given structure without varying the other input parameters, shown in Figure 5. As mentioned earlier, the axial ratios in the Shkarofsky spectrum define different shapes (rods/wings/sheets) of the density structures. A rod-like pattern (for irregularities elongated along the field lines) can be analyzed with different elongation factors in the field line direction (similarly for sheets/wings).

As seen from Figure 5, a range of axial ratios with elongation factor for rod-like irregularities (1:1:*a*${}_{z}$, *a*${}_{z}$> 1) in *a*${}_{z}$ direction is varied, for sheet-like irregularities (*a*${}_{x}$:1:*a*${}_{z}$, *a*${}_{x}$= *a*${}_{z}$), both *a*${}_{x}$ and *a*${}_{z}$ are varied, and for wing-like irregularities (*a*${}_{x}$:1:*a*${}_{z}$, *a*${}_{z}$> *a*${}_{x}$), the elongation factor in *a*${}_{x}$ direction can be changed. We choose the axial ratio values to cover a broader range of elongation factors for rod-shaped irregularities and the spread for wing-like irregularities. The irregularities with axial ratios representing wing-like structures are analyzed for two F-region events (SAGA1 and SAGA2) and irregularity axial ratios that represent rod-like structures are analyzed for the E-region event (SAGA3). The best-fit values of axial ratios for all three events can be seen in Table 2. We also iterate over the outer scale (*l*_0_) and the irregularity layer thickness ($L_{th}$) parameters. It is found that the scintillation strength increases as the irregularity thickness increases and decreases with the increase in the outer-scale length of the irregularities (figure not shown).

**Figure 7:**
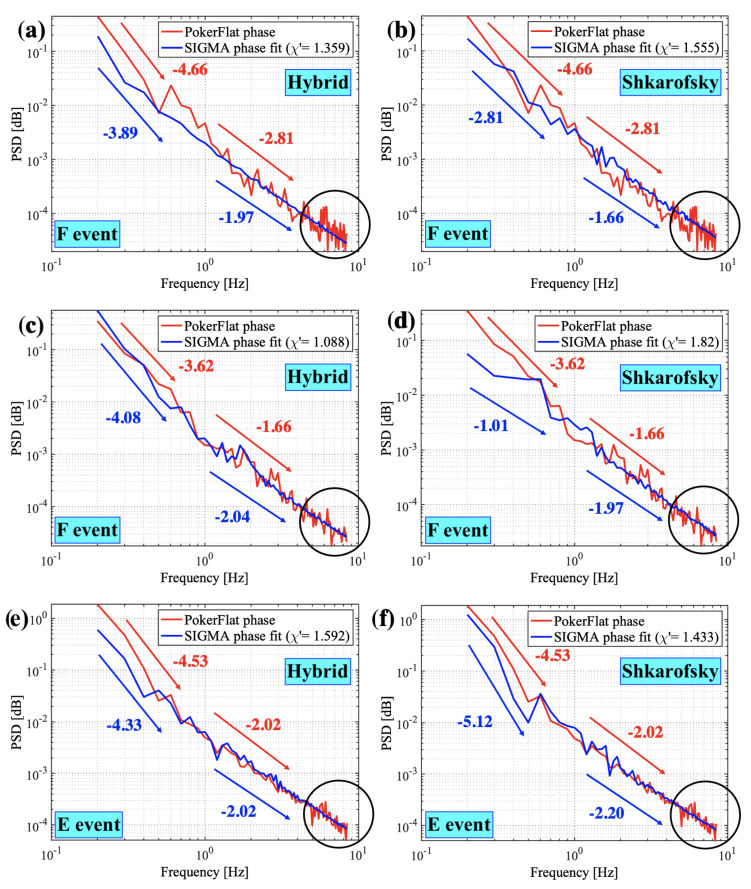
Comparison of observed vs. simulated phase PSD slopes. The left columns (**a**,**c**,**e**) represent the Hybrid spectrum, and the right columns (**b**,**d**,**f**) represent the Shkarofsky spectrum for all three events. The slopes are computed for PSDs both at lower (below 1 Hz) and higher frequencies (above 1 Hz). The part inside the circles is considered noise.

Furthermore, spectral slopes are evaluated using the PSDs extracted from the phase time series. As mentioned in Section 2, the filter cut-off frequency of 0.1 Hz to obtain phase fluctuations from the raw data is a convenient pick for the inverse method with thousands of simulated time series. We compute the slopes of the observed and simulated PSDs to compare the spectral properties of E- and F-region events. We calculate a linearly fitted slope at lower frequencies (0.2 Hz to 0.9 Hz) and higher frequencies (1 Hz to 4 Hz). We are not considering the part inside the circles shown in Figure 7 for slope calculations, assuming it is inside the noise spectrum. The authors would like to acknowledge that the PSD spectrum generally shows frequency-dependent contributions of the refractive vs. diffractive effects; however, the scope of the slope analysis here is to compare the E- and F-region spectral slopes on the ground and relate them to those at the irregularity height without delving much into refractive vs. diffractive discussion. As shown in Figure 7, just like the observed PSDs, even simulated PSDs show the same trend of mostly having steeper (except the one in Figure 7d) spectra at frequencies below 1 Hz and relatively shallower spectra at frequencies above 1 Hz. Such a similar trend in observed and simulated slopes gives us confidence in our methodology. We compare the spectral slopes estimated on the ground to the spectral indices at irregularity height. We found that the spectral slopes on the ground are smaller than the spectral indices at irregularity height. In addition, the spectral indices for E- and F-region events are computed and are shown in Table 2. It shows that the spectral index for the E-region event is less than those for the F-region events, which will be discussed in detail in the next section.

The choice of the number of events we analyze in this work is not only based on the computational constraint to perform a detailed inverse and sensitivity analysis but also based on the availability of the simultaneous measurements from multiple SAGA receivers with at least 30 s long continuous scintillation and either of PFISR, ASI, Swarm, and Defense Meteorological Satellite Program (DMSP) measurements. The detect, classify and hypothesize (DCH) method applied by [36] reveals very few such phase scintillation events. In the following section, we discuss our results in detail and recommend future work on this topic.

## 4. Discussion

In this section, we discuss the morphological features of the irregularities at E- and F-region altitudes that caused the signal fluctuations for the events under study. We also explain the spectral characteristics of E- and F-region irregularities deduced using the two spectral models. Furthermore, we describe the outcomes of our sensitivity analysis performed by varying the irregularity axial ratios and how those relate to the possible shapes of irregularities (rods/wings/sheets) responsible for the signal fluctuations.

### 4.1. Morphology of the Irregularity Structures at E and F Heights

Due to high field-parallel conductivity, electron density irregularities in the ionosphere are expected to be more elongated along the magnetic field lines than across the field lines in the high latitude regions [19]. As discussed earlier, the irregularities can have rod-like, sheet-like, and wing-like anisotropic structures, depending upon the formation mechanism and its location (auroral oval, cusp, and polar cap) [18].

Our findings on the morphology of irregularities responsible for the events mentioned in Figure 1 can be summarized as follows:

- The axial ratios estimated using our inverse analysis indicate that rod-like irregularity structures are more prominent for the E-region event (SAGA3 E-event from Table 2).
- Wing-type irregularity structures are likely responsible for the F-region phase fluctuations for both events (SAGA1 and SAGA2 F-events from Table 2).
- Additionally, the sensitivity analysis shown in Figure 5 support the results that the rod-like irregularities are responsible for E-region phase fluctuations and wing-like irregularities for F-region phase fluctuations.

In order to analyze scintillation effects at a particular location, geometric factors must be considered. In magnetically driven plasma, charged particles move much more freely along the ambient magnetic field lines than across field lines. It leads to field-aligned anisotropic irregularity structures. Anisotropy is characterized by the axial ratio of scale length along the magnetic field to that across the field. The scintillation-producing irregularities generated from diffuse and discrete aurora show a possibility of two different irregularity formations, field-aligned circular and field-aligned elliptical columns [42]. The field-aligned circular irregularities can be related to rod-like structures stretched along the magnetic field lines and isotropic in the other two directions. The field-aligned elliptical structures are related to wing/sheet-like structures, which have an elongation factor in the magnetic east–west direction and the magnetic field-aligned direction. The origins of F-region scintillations are more likely irregularities that are of the form of field-aligned-elliptical structures (wings/sheets) rather than field-aligned-circular structures (rods) [42]. Usually, irregularity structures of wing type are intermixed with those of sheet type. Sheet-like structures are observed during nighttime particle precipitation in the auroral oval at F-region altitudes [19,42,43,44]. The anisotropy patterns may be related to the auroral zone dynamics, such as electron precipitation and plasma instabilities [45]. The nonlinear evolution of E×B drift instability best explains the anisotropy of sheet-like irregularity structures at F-region altitude in the auroral region [22]. Furthermore, the auroral E-region irregularity morphology is related to the current flows in the electrojet that causes field-aligned structures resembling rod-like irregularities [25,26]. A similar irregularity is found in the auroral E-region associated with the ground-back-scattered echo propagated via sporadic E reflection [46]. The field-aligned rod-like structures in the E-region could possibly be formed from the polarization electric fields that map along the geomagnetic field over mid-latitudes [47] and references therein. Precipitation of low energy electrons at F-layer heights produces the F-region irregularities, and that of high energy electrons causes irregularities at E-layer heights [11]. The source of F-region irregularities over Poker Flat was found to be the auroral blobs which were latitudinally and longitudinally stretched, resembling the sheet/wing-like structures [19]. It is further mentioned by [44] that the origin of the source of these auroral blobs could be either polar cusp precipitation or the latitudinally localized precipitation from the inner boundary of the plasma sheet. These were localized but resembled more sheets than rods, and our results from F-region events concur with this morphology. These prior studies support our results, where we found more wing-like irregularities in the F-region and rod-like irregularities in the E-region at auroral latitudes. We observed that the E-region irregularities are produced by hard precipitation of highly energetic electrons along the magnetic field lines resembling rod-like structures.

**Figure 8:**
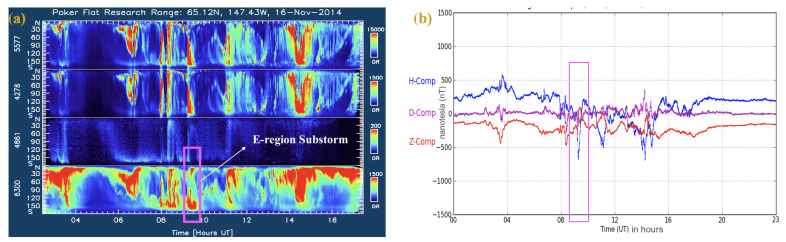
(**a**) Keograms of auroral brightness for E-region (SAGA3) event emitted by O, ${N_{2}}^{+}$, H, and O at wavelengths of 557.7, 427.8, 486.1, and 630.0 nm, respectively. (**b**) The variations of the local magnetic components (pink box highlights the scintillation time).

The E-region event selected in this study is associated with a substorm activity with the electron energies favorable for hard particle precipitation, as shown in Figure 8. The scintillation activity observed in the SAGA3 event at E-region height is more likely to be correlated with the auroral activity (as shown in Figure 2d and Figure 8a) and with the local magnetic field variations (as shown in Figure 8b) occurred near midnight MLT. For E-region cases, we would like to mention that it is not possible to include the contribution of the electric field and the current flows in the current spectral models. Additionally, the more localized SAGA drift estimates (compared to those by ISRs) are not available for E-region cases because the frozen-in drift assumption does not hold at E-region heights. There is a need for a full physics-based model that includes the electric fields, current densities, and strong flow structures.

### 4.2. Variation of the Spectral Index at E- and F-Region Heights

The spectral index is the power-law index for a given irregularity spectrum represented here by either hybrid or Shkarofsky model at the irregularity layer height. Similarly, the spectral slope of a PSD of a time series can give us the power-law index of the scintillation spectrum on the ground. The relationship between the two is discussed below in this subsection. We analyze spectral indices for E- and F-region phase fluctuations using Hybrid and Shkarofsky spectral models. The Shkarofsky spectral model uses a power-law variation of spatial electron number density distribution in all three directions, whereas the Hybrid spectrum uses a power-law (in the plane transverse to the magnetic field line) and Gaussian law (along the field).

Our findings on the spectral characteristics of ionospheric irregularities for the E- and F-region events under study can be summarized as follows:

- We analyzed spectral indices at irregularity layer height for the selected E- and F-region events. We found that the E-region power law index is less than those for the F-region cases (see Table 2);
- Spectral slopes of the PSDs of the phase time series on the ground are steeper at frequencies below 1 Hz compared to those above 1 Hz irrespective of E- or F-region scintillations (see Figure 7);
- The spectral slope of the scintillation spectrum on the ground is less than the spectral index at irregularity layer height.

PSD on the ground is a helpful tool for understanding the relationship between the scintillation spectrum and the irregularity spectrum. The shape of the observed scintillation spectrum plays an essential role in determining the power-law nature of the ionospheric irregularity spectrum [48,49]. The power-law index of the irregularity spectrum at the irregularity layer (3-D spectral index) height is related to the spectral slope of the PSD on the ground (2-D spectral index). The spectral index *p* at the bottom of the irregularity layer reduces to p−1 at the ground level by integrating the 2-D spectrum over the wavenumbers along the ray path [17]. We compute a two-component spectral slope on the ground from the PSDs of the phase time series at frequencies below and above 1 Hz. We would like to mention that although we are not explicitly using two-slope density spectra with p1 and p2 as spectral indices in our inverse analysis, we believe that the spectral models that we use in this study with spectral index *p* could generate a two-slope PSD by covering the irregularity spectra with a wide range of irregularity scale sizes. The linearly fitted two-slope spectra using observed and simulated PSDs are shown in Figure 7. We use these numbers to compare the 2-D spectral index on the ground (derived from scintillation spectra or PSDs) with the 3-D spectral index at the irregularity layer (spectral indices derived from spectral models). Table 2 shows the spectral indices at the irregularity layer height for E- and F-region events. We observed that the spectral slopes of PSDs on the ground at above 1 Hz frequency are less than the spectral slopes at the irregularity layer height for all the events. In other words, the 2-D spectral indices are less than the 3-D ones for both E- and F-region events at the high-frequency regime. Even though this observation does not exactly follow the *p* to p−1 relationship, it agrees with [17] where they mention the spectral index at the bottom of the irregularity layer reduces along the ray path reaching the ground level. We are considering the high-frequency regime here to focus on the contributions from the irregularity towards the diffractive regime for simplicity and acknowledge that the random density distributions would have a significant diffractive component. Two of the three events studied in this paper are phase-only events, meaning amplitude scintillations were negligible. The highest resolution of density structures we use in SIGMA is 100 m at max, which is not enough to resolve Fresnel scale (or below) structures that are important to simulate amplitude scintillations. This occurs because of the tradeoff between the largest simulation box size (50 km) and the smallest scale present in the density distribution. The intense computation inside SIGMA limits maximum resolution in SIGMA. This will be explored in future work, especially as we do studies with coupled GEMINI-SIGMA models. In addition, we compare the spectral indices observed at E- and F-region irregularity heights. As mentioned earlier, the spectral index values computed in the literature for F-region irregularities in the high-latitude zone vary from 2.5 to 4.5 [17,28,50] and for E-region, they vary from 1.5 to 3.2 [29]. Table 2 compares E- and F-region spectral indices. The spectral index values we obtained for both E- and F-region events are in this range. We observed that the spectral index at E-region (3 and 3.5) irregularity height is less than the spectral indices at F-region (4 and 4.5) irregularity height. The behavior of spectral indices may depend on the irregularity dynamics at E- and F-region heights in the auroral region. The steepening in spectra caused by the auroral acceleration due to E-region conductivity on F-region structures can produce a one-dimensional spectral index of ∼3 for irregularity scale size (λ)<1 km [51]. Furthermore, the spectral index variation also depends on the intensity of GPS fluctuations. The spectral characteristics of equatorial F-region irregularities measured from satellite measurements made by [52] show that the spectral index *p* may decrease with an increase in perturbation strength of signal fluctuations. Although this was an equatorial study, it relates to scintillation strength and spectral index. We observed a decreased spectral index for increased scintillation strength in the E-region event. The spectral features in terms of spectral indices of E- and F-region irregularities found using Hybrid and Shkarofsky spectral models are distinguishably different. However, properly quantifying the effect of different source mechanisms and the strength of background density on the ground spectra is beyond the scope of this work. A high-resolution plasma physics model Geospace Environmental Model for Ion-Neutral Interactions (GEMINI) [53,54,55] coupled with SIGMA can be used for more realistic constraints on precipitation, multiscale flow, and density structures to obtain a better estimate of plasma dynamics for auroral events and study their effects on scintillation in order to investigate it further.

## 5. Conclusions

In this work, we use a 3-D propagation model coupled with GNSS scintillation observations and an inverse method to analyze the spectral and morphological features of E- and F-region irregularities in the auroral region at Poker Flat. The EM wave propagation model SIGMA simulates a GPS signal propagated from a moving satellite to the ground through a phase screen. The phase screen is the spatial electron number density distribution characterized using one of the two spectral models: Hybrid and Shkarofsky. We perform an inverse method analysis to derive the parameters describing the ionospheric irregularities by estimating the best fit of model outputs to GPS observations. A sensitivity analysis is further carried out to analyze how sensitive the input parameters are to the model outputs for individual cases. We looked at SAGA1 (F-region), SAGA2 (F-region), and SAGA3 (E-region) events during geomagnetically active times. The E-region event is associated with a substorm activity with electron energies favorable for hard precipitation. For our cases, the precipitation of low-energy electrons produces wing/sheet-like irregularities at F-region heights, whereas high-energy electrons produce rod-like irregularities at E-region heights in the auroral region.

We highlight the major findings of this study below:

1. The axial ratio analyzed for the E-region event reveals that the irregularities are more elongated along the magnetic field lines having rod-like structures. On the other hand, the F-region irregularities have wing/sheet-like structures with irregularity axial ratios extending both along and across the field lines.
2. The spectral indices analyzed at E- and F-region irregularity heights show that the E-region spectral index is less than the F-region spectral indices.
3. Spectral slope analysis compares the slopes on the ground with those at the irregularity layer. We found that the spectral slopes on the ground are less than the slopes at the irregularity layer height for E- and F-region events, consistent with what is expected.

Scintillation in the auroral region has been particularly challenging to understand how and why irregularities form from the fundamental physics point of view. Much of this complexity comes from the fact that models, particularly at E-region heights, need to account for the simultaneous occurrence of strong flow structures, field-aligned currents, electric fields, and a broad spectrum of energy fluxes that often occur in a single auroral structure. Using detailed inverse and sensitivity analysis, our work in this paper derives that E- and F-region irregularities are distinct in morphological and spectral perspective; they have specific preferred structures (rod vs. wing); and spectral indices. We believe that different source mechanisms, background density strength, and drifts play an important role in this distinction. As follow-up research, we plan to include the effects of auroral plasma dynamics for more realistic constraints on the auroral structures, thereby investigating E-region irregularity characteristics and their effects on radio communication using a physics-based plasma model GEMINI coupled with SIGMA. 

## Figures and Tables

**Table 1:** A comparison of observed and simulated values of the propagation parameters. $H_{iono}$ is 120 km (350 km) with a thickness of 10 km (50 km) for the E-region (F-region) event. We consider an outer scale of 15 km for all events. The drift direction estimates are measured counter-clockwise from the geomagnetic south. The SAGA drift estimates are shown in boldface.

	SAGA1—F Event		SAGA2—F Event		SAGA3—E Event	
Parameter	Observed	Simulated	Observed	Simulated	Observed	Simulated
**Hybrid Spectrum**						
$N_{e}$ (el/m^3^)	(0.2–0.4) × 10^12^	0.2 × 10^12^	(0.7–1.1) × 10^12^	0.75 × 10^12^	(0.7–1.1) × 10^12^	1.05 × 10^12^
$|v_{d}|$ (m/s)	1000–1500 **(1500)**	1550	450–700 **(500)**	550	500–800	500
$∠v_{d}$	130${}^{\circ }$–200${}^{\circ }$ **(140**${}^{\circ }$**)**	160${}^{\circ }$	290${}^{\circ }$–360${}^{\circ }$ **(290**${}^{\circ }$**)**	300${}^{\circ }$	130${}^{\circ }$–200${}^{\circ }$	160${}^{\circ }$
**Shkarofsky Spectrum**						
$N_{e}$ (el/m^3^)	(0.2–0.4) × 10^12^	0.27 × 10^12^	(0.7–1.1) × 10^12^	1.05 × 10^12^	(0.7–1.1) × 10^12^	0.74 × 10^12^
$|v_{d}|$ (m/s)	1000–1500 **(1500)**	1500	450–700 **(500)**	500	500–800	825
$∠v_{d}$	130${}^{\circ }$–200${}^{\circ }$ **(140**${}^{\circ }$**)**	140${}^{\circ }$	290${}^{\circ }$–360${}^{\circ }$ **(290**${}^{\circ }$**)**	290${}^{\circ }$	130${}^{\circ }$–200${}^{\circ }$	130${}^{\circ }$

**Table 2:** The spectral parameters of SIGMA obtained using inverse analysis for all three events.

	SAGA1 F—Event		SAGA2 F—Event		SAGA3 E—Event	
Parameter	Hybrid	Shkarofsky	Hybrid	Shkarofsky	Hybrid	Shkarofsky
Axial Ratio	5	*a*${}_{x}$ = 2, *a*${}_{y}$ = 1, *a*${}_{z}$ = 10	5	*a*${}_{x}$ = 2, *a*${}_{y}$ = 1, *a*${}_{z}$ = 10	6	*a*${}_{x}$ = 1, *a*${}_{y}$ = 1, *a*${}_{z}$ = 10
		**Wing-like**		**Wing-like**		**Rod-like**
Spectral Index	4.5	4.5	4	4.5	3	3.5

**Table 3:** The inverse analysis performed for three different irregularity structures (rods/wings/ sheets) for each of the three events. Underlined are the best fits.

	SAGA1 F—Event	SAGA2 F—Event	SAGA3 E—Event
Wings	$\chi^{\prime }=1.55$	$\chi^{\prime }=1.82$	$\chi^{\prime }=4.8$
Sheets	$\chi^{\prime }=7.21$	$\chi^{\prime }=5.87$	$\chi^{\prime }=5.2$
Rods	$\chi^{\prime }=3.2$	$\chi^{\prime }=6.67$	$\chi^{\prime }=1.43$

## Data Availability

The SAGA observed data and the SIGMA simulated files for the scintillation events that are identified and analyzed in this work are available as https://doi.org/10.5281/zenodo.6621888 (accessed on 15 November 2022) at https://zenodo.org/badge/latestdoi/500990398 (accessed on 15 November 2022). The authors acknowledge the use of SAGA data that is publicly available at http://apollo.tbc.iit.edu/~spaceweather/live/?q=SAGA (accessed on 15 November 2022) and PFISR data available at https://isr.sri.com/madrigal/cgi-bin/gSimpleUIAccessData.py? (accessed on 15 November 2022). The magnetometer data were obtained from the Geophysical Institute Magnetometer Array online service http://magnet.gi.alaska.edu/, (accessed on 19 April 2022).

## Supplementary Materials

The following supporting information can be downloaded at: https://doi.org/10.5281/zenodo.7630323 accessed on 15 November 2022, Figures: Height and thickness sensitivity plots.

## Abbreviations

The following abbreviations are used in this manuscript:

GPS	Global Positioning System
GNSS	Global Navigation Satellite System
GDI	Gradient Drift Instability
KHI	Kelvin-Helmholtz Instability
MLT	Magnetic Local Time
PSD	Power Spectral Density
